# Dual growth factor-modified microspheres nesting human-derived umbilical cord mesenchymal stem cells for bone regeneration

**DOI:** 10.1186/s13036-023-00360-w

**Published:** 2023-07-10

**Authors:** Wenzhi Song, Lanlan Zhao, Yuqi Gao, Chunyu Han, Shengrui Gao, Min Guo, Jianfei Bai, Liqiang Wang, Wanzhong Yin, Feng Wu, Peibiao Zhang

**Affiliations:** 1 Department of Stomatology, China-Japan Union Hospital, Jilin University, Changchun, 130031 PR China; 2 Department of Otorhinolaryngology, First Clinical Hospital of Jilin University, Changchun, 130021 PR China; 3 Key Laboratory of Polymer Ecomaterials, Changchun Institute of Applied Chemistry, Chinese Academy of Sciences, Changchun, PR China; 4 Department of Ophthalmology, Third Medical Center, Chinese PLA General Hospital, Beijing, 100853 PR China; 5 Foshan Hospital of Traditional Chinese Medicine/Foshan Hospital of TCM, Foshan, China

**Keywords:** Osteon-like micromodule, Growth factor, Ratio optimization, Stem cell therapy, Bone repair

## Abstract

**Background:**

Modular tissue engineering (MTE) is a novel “bottom-up” approach that aims to mimic complex tissue microstructural features. The constructed micromodules are assembled into engineered biological tissues with repetitive functional microunits and form cellular networks. This is emerging as a promising strategy for reconstruction of biological tissue.

**Results:**

Herein, we constructed a micromodule for MTE and developed engineered osteon-like microunits by inoculating human-derived umbilical cord mesenchymal stem cells (HUMSCs) onto nHA/PLGA microspheres with surface modification of dual growth factors (BMP2/bFGF). By evaluating the results of proliferation and osteogenic differentiation ability of HUMSCs in vitro, the optimal ratio of the dual growth factor (BMP2/bFGF) combination was derived as 5:5. In vivo assessments showed the great importance of HUMSCs for osteogneic differentiation. Ultimately, direct promotion of early osteo-differentiation manifested as upregulation of Runx-2 gene expression. The vascularization capability was evaluated by tube formation assays, demonstrating the importance of HUMSCs in the microunits for angiogenesis.

**Conclusions:**

The modification of growth factors and HUMSCs showed ideal biocompatibility and osteogenesis combined with nHA/PLGA scaffolds. The micromodules constructed in the current study provide an efficient stem cell therapy strategy for bone defect repair.

## Introduction

The underlying goal of bone tissue engineering is the construction of biofunctional structures that regulate the proliferation, migration and differentiation of stem cells by incorporating architectural design concepts into biological materials [1, 2]. Current traditional tissue engineering (TTE) strategies usually use a “top-down” approach [3], where cells are inoculated in a precustomized three-dimensional (3D) scaffold that replaces the entire damaged tissue according to the defective morphology. However, most studies focus on macroscopic constructs that replicate the overall size and shape of the target tissue rather than the complex internal structure Problems such as limited nutrient transport and metabolic barriers within the scaffold hinder the depth of bone tissue regeneration at the center of the scaffold [4].

In recent years, an emerging concept in “bottom-up” microstructure engineering, modular tissue engineering (MTE), has provided new therapeutic strategies [3]. In MTE, small building blocks are used to assemble complex tissue-like structures. Meiling Zhong et al. prepared collagen microspheres as bone-like modules by seeding HUVECs on collagen microspheres containing MG63 and collagenase and constructed a prevascularized bone tissue biomimetic through the assembly of bone-like modules [5]. This bottom-up construction method has a strong biological basis in organisms, where many tissues or organs consist of repetitive microfunctional modules, such as liver lobules, muscle fibers, and other microstructures [6]. Likewise, MTE products have better biomimetic properties than those from traditional methods simulating natural microstructural functional units and assembling macroorganisms. Therefore, modular tissue engineering holds great promise for tissue regeneration therapy.

MTE also includes three components: microscale bioscaffolds, cytokines, and seed cells. Biomaterials for building micromodules require the design of structural features at the microscale while preserving the cellular functions of bone-like microunits. Microspheres can be used as microfolding materials for MTE due to their micron-sized dimensions and good mechanical properties. The relative surface area of microspheres facilitates the nutritional intake of osteon-like microunits [7].

Based on a previous in-depth study of microspheres by our group [8], a novel airflow shear method was developed to prepare polyester polymer microspheres, which could not only scale up the production to the tissue scale, but also control size and uniformity precisely. The as-prepared nanohydroxyapatite and polypropylene cross-ester-ethyl cross-ester nanocomposite microspheres(nHA/PLGA) exhibited a unique surface morphology and good osteogenic ability. They can be employed as potential candidates for bone graft materials for bone regeneration. Moreover, the scaffold in the form of nHA/PLGA microspheres can be injection molded [9] and easily assembled into macroscopic tissues by stacking [10], thus filling defects with a less invasive technique that is less harmful to patients [11]. Due to the rapid degradability of nHA/PLGA microspheres and their osteoinductive ability, the randomly distributed microspheres in the area of bone defects become gradually deformed or even fragmented over time, facilitating in situ regeneration. In addition, the higher relative surface area of microspheres also facilitates the acquisition of nutrients and the discharge of metabolic waste, which effectively improves the efficiency of stem cell survival. Therefore, the as-prepared nHA/PLGA microspheres can serve as MTE scaffolds for growth factor loading and cell delivery.

Bone marrow-derived mesenchymal stem cells (BMSCs) are considered to be the main source of MSCs. They are commonly used as seed cells for TTE. However, they are not always suitable for clinical settings since autologous transplantation is inconvenient to prepare and the expansion time is long. In addition, difficult access, insufficient resources, and ethical issues also affect the clinical effectiveness of MSCs [12]. Human umbilical cord mesenchymal stem cells (HUMSCs) derived from neonatal umbilical cord tissue have the advantages of convenient access, no ethical controversy, strong proliferation ability and low immunogenicity [13, 14]. These are gradually becoming an ideal substitute for BMSCs. As a readily available source of seed cells for bone tissue engineering, HUMSCs should have broad application prospects. However, there are few studies on the osteogenic differentiation of HUMSCs at present, which may be due to their low osteogenic differentiation efficiency and multidirectional differentiation potential.

Growth factors have been shown to be particularly effective at inducing stem cells to differentiate into specific cell lineages [15]. Bone morphogenetic protein 2 (BMP2) is a member of the transforming growth factor-β (TGF-β) superfamily [16]. Sustained delivery of BMP2 leads to strong osteogenic activity and has the potential to directly enhance bone integrity [17]. However, the function of BMP2 alone is relatively unitary considering the complexity of the bone regeneration process. In vivo implanted scaffolds that only modify BMP2 cannot achieve the ideal osteogenic effect, which limits their application in bone tissue engineering therapy. Studies have shown the great importance of the long-term release of low-dose bFGF and BMP2 in early osteogenesis [18, 19]. bFGF can significantly promote the proliferation of stem cells and maintain the characteristics of mesenchymal stem cells [20, 21] and was proven to promote bone regeneration through the Wnt/β-Catenin signaling pathway [22]. The combination of dual growth factors can produce a synergistic effect that may further induce complex cellular interactions to promote new bone formation [23]. However, under nonoptimized conditions, the combined delivery of BMP2 and bFGF reduced the osteogenesis of mesenchymal stem cells [24]. To enhance the synergistic osteogenic effect of these two growth factors, it is necessary to further optimize parameters such as the concentration and ratio of each growth factor.

In the current study, nHA/PLGA microspheres were used as microscaffolds. By modifying the surface with polydopamine (pDA) as an effective adhesion layer, the scaffold could carry dual growth factors (BMP2 and bFGF) and HUMCSs to construct MTE micromodules. Subsequently, by optimizing the ratio parameters of the dual growth factors, the expansion and osteogenic differentiation of HUMSCs in vitro were further analysed. In addition, in vivo experiments were performed to evaluate the bone repair ability of osteogenic microunits loaded with HUMSCs. Finally, the auxo-action of bone osteogenesis and vascularization was confirmed by PCR and tube formation assays. A promising new stem cell therapy strategy is proposed for bone defect repair.

## Methods and materials

Poly lactic-co-glycolic acid**(**PLGA, lactide/glycolide ratio is 75:25, Mn is 10 W) and basic fibroblast growth factor(bFGF) were synthesized by Changchun Institute of Applied Chemistry, Chinese Academy of Sciences (CIAC, China). BMP2 was purchased from Sinobiological (China). HA nanoparticles (nHA) were synthesized as described in our previous study [25]. Analytical grade (99.99% purity) N-Methylpyrrolidone (NMP) was purchased from Sigma − Aldrich. Dulbecco’s modified Eagle medium (DMEM),Dulbecco’s modified Eagle medium nutrient mixture F-12 (DMEM F-12), fetal bovine serum (FBS), and trypsin − EDTA were purchased from Gibco (USA).

### Fabrication of nHA/PLGA

The nHA/PLGA nanocomposite microspheres were prepared by the airflow shearing method, with an optimum concentration of 20 wt% nHA and a microsphere diameter range of 200–350 μm [8]. Briefly, a 25% (w/v) PLGA NMP solution was prepared with a 20 wt% proportion of ultrasonically dispersed nHA. Then, the mixed suspension was injected into the syringe and mounted on the squeezing device set with appropriate speed. The mixed suspension was squeezed out at a set rate. The droplets were uniformly cut by nitrogen gas, forming a large number of small droplets. The droplets rapidly solidified and formed nHA/PLGA microspheres (M) after being picked up by a 60% ethanol aqueous solution. Microspheres of the desired size were then screened using a sieve and set aside for subsequent experiments.

### Surface modification of nHA/PLGA microspheres with polydopamine (pDA)

The polydopamine (pDA) surface coating was prepared as described in our previous study [26]. Briefly, nHA/PLGA microsphere samples were placed in 48-well cell culture plates (Corning Costar, USA), and 1 ml of preconfigured pDA solution (2 mg/ml in 10 mM Tris–HCL, pH = 8.5) was added to each well and placed on a constant temperature shaker at 37 °C for 4 h. Afterward, the samples were removed and placed in a new 48-well cell culture plate and repeatedly rinsed with deionized water to remove the unfastened adherent pDA. The samples were named MD.

### Loading and quantitative analysis of growth factors

In this study, a concentration of 50 ng/ml was chosen for ratio optimization [27, 28]. For the loading of BMP2/bFGF growth factor, different ratios (2:8, 5:5, 8:2) (Table1) of 500 μl mixed solutions as well as two kinds of single-composition solutions were injected into 48-well plates. The different dual growth factor mixture ratios are listed in Table1. These experimental groups were named MB, MF, 2:8, 5:5 and 8:2.

**Table 1:** Different concentrations of the BMP-2/bFGF dual growth factor mixture

GF	MB	MF	2:8	5:5	8:2
BMP-2(ng/ml)	**50**	**0**	**10**	**25**	**40**
bFGF(ng/ml)	**0**	**50**	**40**	**25**	**10**

The amount of immobilization of BMP2 and bFGF was measured indirectly using enzyme-linked immunosorbent assay (ELISA). The amount of surface-immobilized growth factors was derived from the difference between the initial concentration and the postincubation concentration. Finally, the respective adhesion rate of BMP2/bFGF in the combined group of compound growth factors was calculated. All ELISA experiments were performed according to the manufacturer's protocol. Sample absorbance was measured at 450 nm using a spectrophotometer, and 540 nm was used for λ correction.

### Characterization of microspheres

The general morphology changes of MD were observed. The surface morphology of the microspheres in the wet state was observed by a stereomicroscope. Because the microspheres are not transparent, it is difficult for the light source of the commonly used optical microscope to penetrate the microspheres, and the microspheres become deformed in the freeze-dried state. Therefore, the micromorphological changes of MD were observed by field emission scanning electron microscopy (SEM). The MD was glued on the stage with conductive adhesive, and its microstructure was observed under a scanning electron microscope (SEM) after uniformly spraying gold. In the meantime, the element distribution was analyzed by EDS mapping.

Fourier transform infrared spectroscopy (FTIR, Perkin Elmer, FTIR-2000) was used to evaluate the chemical structure in the wavenumber region from 400 to 4000 cm^−1^. The X-ray diffraction (XRD) patterns of microspheres were determined by XRD (Bruker Co., Germany) at 40 kV and 20 mA in a 2 h range of 10–60° with a step of 0.03°. Thermogravimetric analysis was carried out using TGA. The conditions were as follows: a heating rate of 10 ℃/min from 40 to 800℃ under a nitrogen atmosphere.

X-ray photoelectron spectroscopy (XPS) was performed with Thermo Scientific K-Alpha using monochromatic Al Kα radiation(hν = 1486.6 eV). The elemental composition on the sample surface was obtained by a standard quantitative XPS analysis of survey spectra acquired at a pass energy of 100 eV in the binding energy range of 0–1400 eV.

### Construction of MTE units

HUMSCs were cultured in F12 medium containing 10% fetal bovine serum, in a 5% CO_2_, 37 °C cell culture incubator, with fluid changes every two days. pDA/nHA/PLGA nanocomposite microsphere samples were sterilized with 75% ethanol for 30 min before loading growth factors and growing HUMSCs, followed by sterilization in a UV sterilizer for 2–3 h. After sterilization, the samples were repeatedly rinsed on an ultraclean table with PBS 3 times and placed in 48-well cell culture plates. The different ratios of growth factor solutions were sterilized by filter heads and incubated in 48-well plates for 12 h at 4 °C.

Construction of osteo-like micromodules (MC): HUMSCs (1.5 × 10^4^ cells/ml) were inoculated onto microsphere scaffolds in 48-well plates, and cell carriers loaded with growth factors and HUMSCs were used as osteon-like micromodules (MC). The cell culture medium was changed every 2 days. Three parallel samples were set up for each group of all in vitro cytology studies, and each group of experiments was repeated 3 times.

### Cell proliferation assay

The effect of MD modified with different ratios of the BMP2/bFGF complex growth factor on the proliferation ability of HUMSCs was assessed by the CCK-8 method. HUMSCs were inoculated at a density of 2 × 10^4^ cells per well on microcarrier samples (approximately 6 mg per well) in 48-well plates and cultured for 1, 3 and 7 days. At each defined time point, 15 μl CCK-8 solution was added to each well. After 2 h of incubation, 200 μl of medium was transferred into a 96-well plate for measurement. The absorbance was measured at 450 nm using a multifunctional microplate scanner (Tecan Infinite M200). The single growth factor group and MD were used as controls, and the results were averaged for three parallel samples.

### Cell adhesion assay

Cell adhesion was assessed by calcium xanthophyll AM (green live cell) staining. HUMSCs (2 × 10^4^ cells/well) were inoculated onto microspheres in 48-well plates and cultured in a cell incubator at 37 °C and 5% CO2. After 4 days, HUMSCs on microspheres were fixed with 4% paraformaldehyde and stained with calcein AM. Dead cells (red) were stained with PI for 2 min. Images were taken by a fluorescence inverted microscope (TE2000U, Nikon).

HUMSCs were seeded on different microspheres in 24-well plates for 4 days. The cells were fixed in 4% PFA for 30 min. and immersed in Immunol Staining Blocking Buffer (Beyotime, China) for 1 h. After incubating with the corresponding primary antibodies, proportionally diluted in 5% bovine serum albumin (BSA, Solarbio), at 4 °C overnight, the samples were incubated with second antibodies for 2 h. Furthermore, the nuclei of the cells were stained with DAPI (Solarbio, China) for 2 min. Finally, images were captured by fluorescence microscopy (Nikon, Japan).

### Alkaline phosphatase (ALP) activity assay

The effect of MD modified by different ratios of dual growth factors on the osteogenic differentiation ability of HUMSCs was assessed using the ALP assay. HUMSCs were inoculated in 48-well cell culture plates at 2 × 10^4^/well and cultured for 7 days. After the cells were cultured to the corresponding time point, the well plates were placed on ice boxes. The culture medium was aspirated and discarded. PBS solution was gently used to wash the microspheres three times. Two hundred microliters of Western and IP cell lysis solution (without inhibitors) was added to each well and gently blown. Then, the freeze-thawing was repeated twice to fully lyse the cells adhering to the surface of the microspheres. A new 96-well plate was prepared. Fifty microliters of lysis solution and 50 µl of pNPP reagent were added to each well. The plate was incubated at 37 °C for 30 min, and the absorbance at 405 nm (OD405) was read using a multifunctional microplate scanner (Tecan Infinite M200). The total protein contained in the lysate was also measured using the BCA kit. First, the BCA working solution was prepared by mixing the A and B solutions in a 50:1 volume ratio, and 200 µl of BCA working solution was added to each well of a 96-well plate. Then, 20 µl of the abovementioned cell lysate was added to each well. The plate was incubated at 37 °C for 30 min. A Tecan Infinite M200 was used to read the absorbance at 562 nm (OD562). The corresponding quantitative assessment of ALP was calculated according to the OD405/OD562 formula.

### Alizarin red staining for calcium deposition

The in vitro mineralization capacity of HUMSCs after 7 days of culture was investigated using the alizarin red staining (ARS) method. Calcium deposition was quantified using the cetylpyridinium chloride (CPC) assay. Microcarriers were cocultured with cells as described above and incubated to the corresponding time point. The medium was aspirated and discarded, and the cells were gently washed 3 times with deionized water and fixed in 4% paraformaldehyde solution for 30 min at room temperature. Each well was incubated with ARS reagent (submerged microspheres) for 30 min at room temperature and gently washed 3 times using deionized water. For the quantitative assay, 500 µl of 10% CPC solution was added to each well and incubated for 1 h at room temperature. One hundred microliters of each well was aspirated and transferred to a 96-well plate. The absorbance was measured at 540 nm (OD540) using a multifunctional microplate scanner (Tecan Infinite M200).

### Construct assembly for in vivo bone repair study

Based on the results of in vitro experiments, the optimal ratio of the dual growth factors (BMP-2/bFGF) was used to construct osteon-like micromodules, which were further assembled into macroscopic osteon-like tissue structures with HUMSCs. The micromodules were cultured in vitro for 3 days and implanted into a living model of rat cranial bone defects. Each modified layer of the construction process was used for comparison.

### In vivo model of bone defects on the skull of rats

A total of 24 8-week-old male Sprague‒Dawley rats weighing 200–250 g were used for in the vivo study, with each used to construct 2 cranial critical bone defect models. These 48 models in total were randomly divided into 2 major groups according to two time points of 4 weeks and 8 weeks. According to the experimental materials, there were 5 parallel samples in each random group: Group 1-MC; Group 2–5:5; Group 3-MD; Group 4-M; and Group 5-B (*n* = 4). The in vivo model was created by removal of 5-mm circular bone on both sides of the sagittal suture on each rat's skull using a circular grinding drill. Preprepared sterile material was implanted into the defect area without any fixation. The wounds were then gently closed with surgical sutures. After surgery, the rats were fed at the Institute of Laboratory Animal Research, Jilin University. All rats were injected intramuscularly with sodium penicillin, 200,000 units per rat, for 5 days. All surgical wounds healed well, and there were no postoperative complications. All surgeries were performed according to the protocol of the Institutional Animal Care and Use Committee of Jilin University and in accordance with the Guide for the Care and Use of Laboratory Animals published by the National Academy of Sciences.

### Micro-CT examination

At week 8, the rats were sacrificed, and the rat head specimens were immersed in 4% paraformaldehyde solution and set aside. The specimens were then scanned by microCT (microCT80, Scanco Medical, Bassersdorf, Switzerland) at a resolution of 10 µm (80 kV, 100 µa) followed by offline reconstruction (grayscale values: 0–0.075). Three-dimensional reconstructions and measurements were acquired to assess bone volume (BV), trabecular thickness (Tb.Th), trabecular separation (Tb.Sp), trabecular number (Tb.N), trabecular pattern factor (Tb.Pf), and bone surface (B.S.). Bone area was calculated using Photoshop CS6 software (Adobe system), and then regenerated bone volume (BV)/total volume (TV) was calculated. For TV, a fixed value sufficient to cover the fully regenerated bone position was selected and was adjusted equally for all samples.

### Histological staining

Animals were sacrificed at different time points, and the implants were removed. Specimens were first fixed in 4% phosphate-buffered paraformaldehyde for 12 h. Immunofluorescence (IF) and histological analyses were performed after µCT scans and decalcified in 10% ethylenediaminetetraacetic acid (EDTA) at 4 °C for 4 weeks. The solution was changed every two days. Prior to staining, samples were refixed, dehydrated in ethanol and embedded in paraffin. The sections were then cut longitudinally into 5-μm-thick sections. To observe the formation of the vascular network and cellular distribution in the macroscopic tissue structure, sections were stained with hematoxylin and eosin (HE) and Masson’s trichrome to observe the overall tissue morphology. For histology, sections were stained with Sirius red and examined microscopically in the presence of polarized light. For immunofluorescent labeling studies, the following primary antibodies were used: anti-ALP (1:400, Servicebio GB11527), anti-osteocalcin (OCN) polyclonal antibody (1:100, Servicebio GB11233) and anti-CD31 (1:500, Servicebio GB11063-2). Stained fluorescent images were observed by fluorescence microscopy and scanned for photographs (*n* = 5).

### Osteogenic-related gene expression

Total RNA was extracted using TRIzol reagent (Invitrogen, Carlsbad, CA, USA), and RNA quality was examined using a NanoDro2000c spectrophotometer (Thermo Scientific, Waltham, USA) according to the manufacturer’s instructions. Subsequently, the mRNA was converted to cDNA using a ReverTra Ace® qPCR RT Kit (Toyobo, Japan). RT-PCR was carried out using a CFX Connect Real-Time PCR Detection System (BIO-RAD, USA). The relative amount of gene transcripts was normalized to the GAPDH level. Data were analyzed and quantified using 2 − △△Ct. The primers used in the present study were designed and synthesized by RiboBio (Guangzhou, China). The sequences of the primers are shown in Table2.

**Table 2:** Sequence of the oligonucleotides for real-time PCR

Gene	Sequence (5´ → 3´)
**Runx-2**	**F 5’-CAGCCCCAACTTCCTGTG-3’**
	**R 5’-CCGGAGCTCAGCAGAATAAT-3’**
**COL-1**	**F 5’-CACAGAGGTTTCAGTGGTTTGG-3’**
	**R 5’-GCACCAGTAGCACCATCATTTC-3’**
**OCN**	**F 5’-CCACCGAGACACCATGAGAG-3’**
	**R 5’-TCACCCAACTCGTCACAGTC-3’**
**GAPDH**	**F 5’-CTCCTGTTCGACAGTCA-3’**
	**R 5’-CCATGGTGTCTGAGCGATGT-3’**

### Tube formation assay

A tubule-forming assay was used to assess angiogenesis of differently treated HUVECs. HUVECs were cultured in 48-well plates (2 × 10^4^/well) at 37 °C and 5% CO2 for 4 days. Each day, the culture medium of HUVECs was replaced by medium immersed in the following scaffolds: M, MD, 5:5, MC. Matrigel (50 μL, BD Biosciences, San Jose, CA, USA) was placed in individual wells of a 48-well plate and allowed to polymerize at 37 °C for 30 min. Treated HUVECs were inoculated at a density of 2 × 10^4^ cells/well for 6 h. The results were observed using a phase-contrast microscope, and the microscopic fields were photographed in three images for each group. The images were analyzed using Image J software (Angiogenesis package). The number of segments, number of nodes, and total segment length of the tubules were calculated for quantitative analysis of angiogenesis.

### Statistical analysis

Independent and replicated experiments were used to analyze the statistical variability of the data via one-way analysis of variance, with *p* < 0.05 being statistically significant.

## Results and discussion

### Structural characterization of nHA/PLGA microspheres and pDA modification

**Fig. 1:**
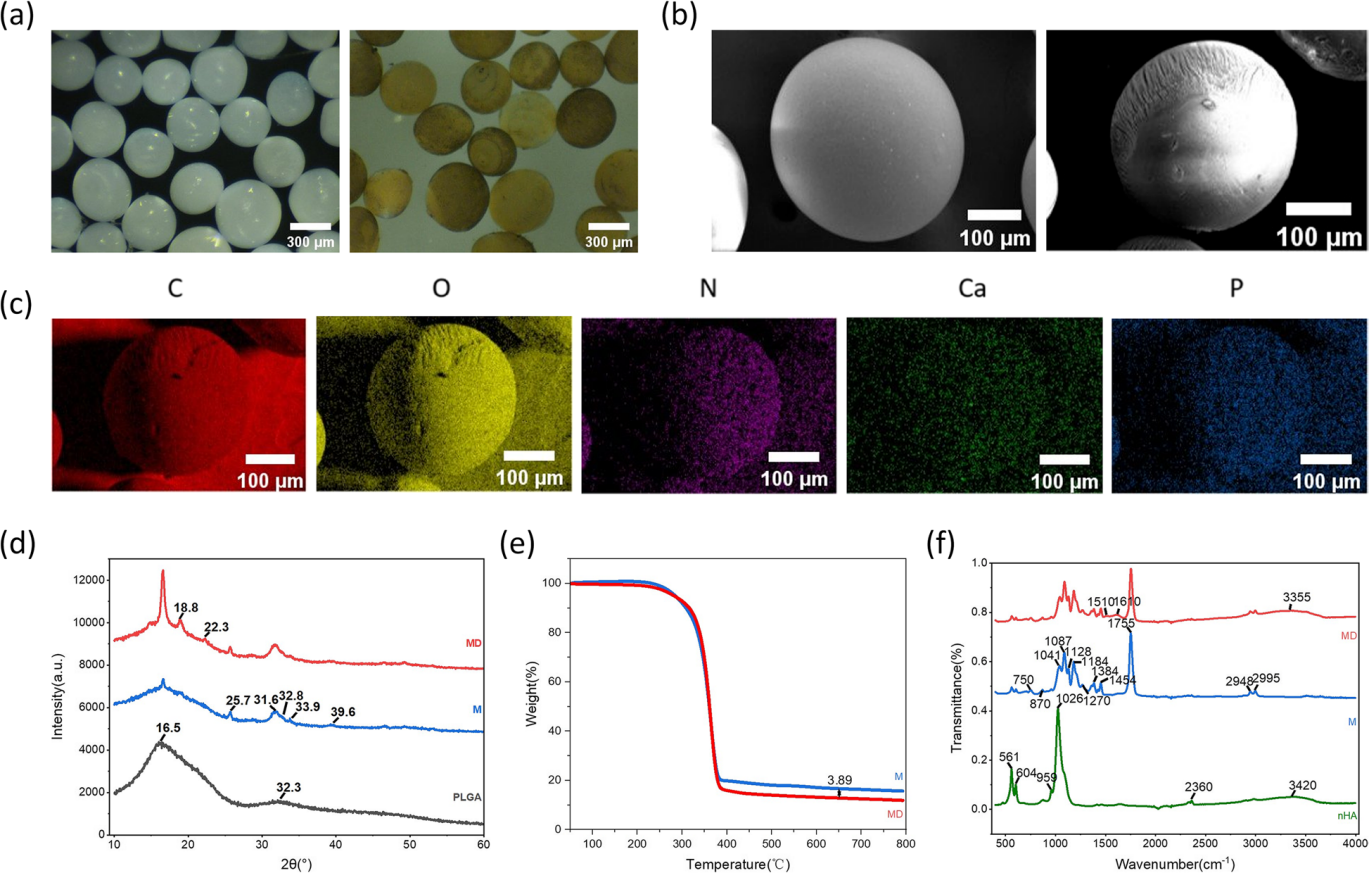
**a** Digital images of nHA/PLGA(left) and pDA/nHA/PLGA(right) nanocomposite microspheres. Scale bar: 300 μm. **b** SEM images of nHA/PLGA nanocomposite microspheres before(left) and after(right) modification with polydopamine. Scale bar: 100 μm. **c** EDS indicating chemical elements of polydopamine-modified nHA/PLGA microspheres. Scale bar: 100 μm. **d** XRD of pDA/nHA/PLGA, nHA/PLGA, PLGA **e** TGA of pDA/nHA/PLGA, nHA/PLGA **f** FTIR spectra of pDA/nHA/PLGA, nHA/PLGA, nHA

Biodegradable polymer microspheres have attracted great attention in the field of tissue regenerative medicine. They are widely used as temporary three-dimensional cell culture scaffolds and drug delivery vehicles in tissue engineering therapy [29, 30]. Therefore, nHA/PLGA microspheres were optimized for the preparation process and investigated for potential applications in bone tissue engineering according to previous research by our group. It was found that in vitro, nHA/PLGA microspheres with high nHA content effectively enhanced the proliferation and osteogenic differentiation of MC3T3-E1 cells. In a mouse cranial defect model, microspheres with nHA contents of 20 w% and 40 w% induced faster mineralization and new bone formation processes, indicating that nHA/PLGA microspheres with high nHA content have potential applications in stem cell expansion and tissue engineering [8]. In this study, 20 wt% nHA/PLGA microspheres were used as microscaffolds for MTE. The morphology of the nHA/PLGA microspheres before and after surface modification with pDA was characterized by stereomicroscopy and SEM. The prepared nHA/PLGA microspheres were milky white, spherical with uniform particle size and good dispersion, and the surface was relatively smooth. In contrast, the surface of nHA/PLGA microspheres modified with polydopamine (MD) became rougher, and the color changed from white to brownish yellow (Fig. 1a). This indicated that the surface of the microspheres was uniformly coated with pDA.

In the SEM images, (Fig. 1b), the nHA/PLGA microspheres showed an “acorn”-like appearance, with a smooth and a dense side and a rough and loose side. The rough side was formed due to the collision between the droplet and the collection fluid during microsphere formation, and the smooth side was formed via the escape of NMP from the droplet. This unique morphology provides a large surface area and facilitates nutrient transport and oxygen diffusion. While the pDA coating adhered to the surface, it could be observed under microscopic magnification that the surface was relatively rough compared with that of nHA/PLGA microspheres. The results are consistent with previous studies by others [31]. The elemental compositions of the microspheres were analyzed by means of EDS mapping. The results (Fig. 1c) indicated that the microspheres were composed of C, O, N, Ca, and P. C and O are mainly derived from PlGA and polydopamine, and Ca and P elements are mainly derived from n-HA. N was observed in the EDS mapping of pDA/nHA/PLGA microspheres, which indicated the adherence of the pDA coating. The TGA results (Fig. 1e) demonstrated the difference in the n-HA content of the two groups, calculated at approximately 3.8%, which showed the components of pDA.

The phase composition analysis by XRD is depicted in Fig. 1d. The characteristic diffraction peaks of PLGA were found at 2θ = 16.5° and 32.3°. Similarly, nHA displayed major characteristic peaks at 2θ = 25.7°, 31.6°, 32.8°, 33.9°, and 39.6°. These characteristic peaks were found in the pDA/nHA/PLGA group, implying the incorporation of nHA and PLGA in the composite microspheres. Two other peaks were observed at 2θ = 18.8° and 22.3°, which possibly indicated the presence of pDA. Furthermore, the compositions of the microspheres were analyzed based on FTIR spectra (Fig. 1f). Peaks of nHA at approximately 561, 604, and 1026 cm-1 were assigned to PO_4_ bonds, and that at 3420 cm^−1^ was assigned to OH bonds, which are typically present in such substances. Thus, peaks at the same wavenumber in the other two groups suggest the existence of n-HA. Between the two microsphere groups, several characteristic peaks we found at similar locations. The stretching vibration absorption peaks generated by the ACH bonds in the spectrum of PLGA occurred at 2948 and 2995 cm^−1^. The carbonyl and C–O–C stretching peaks were at approximately 1755 and 1087 cm^−1^, respectively. The peaks located at 1041 cm^−1^ were assigned to the C–CH3 stretching vibrations. Likewise, the peak at approximately 1450 cm^−1^ was attributed to C–H stretching and was observed in both microsphere spectra. These results indicate the successful addition of nHA nanoparticles to the nHA/PLGA microspheres. In terms of the pDA spectrum, the peaks at 1510 cm^−1^ were related to the bending vibration peak of N–H in the benzene skeleton, and 1610 cm^−1^ was ascribed to the C = C backbone and N–H shear vibration in the polydopamine benzene ring [32]. These results indicated that n-HA and PGCL were the main components of the microspheres prepared by this method without other impurities or residue of NMP. In addition, absorption peaks at 1510 and 1610 cm^−1^ appeared in the pDA spectra, suggesting successful pDA modification on the surface of the microspheres.

In recent years, surface immobilization of growth factors on synthetic substrates mediated by polydopamine has been more widely used in tissue engineering applications. Polydopamine is a natural melanin that is biocompatible and does not cause immune rejection [33, 34]. The catechol moiety in its chemical structure has superb adhesion properties for a wide range of material surfaces. It has been applied to improve the surface properties of a variety of organic and inorganic materials, such as metals, ceramics, semiconductors, and fibers [31, 35]. The formation of polydopamine layers is attributed to the polymerization of catechol and amine groups in dopamine in a slightly alkaline environment [36], and studies have shown that Schiff base reactions or Michael addition between the sulfhydryl or amine groups of biomolecules. The catechol/quinone groups of the polydopamine coating matrix may be responsible for the immobilization of biomolecules on the surface of materials [31, 37]. The electrostatic interaction, coordination bonds and hydrogen bonds make it possible to bond with non-specific bioactive substances and release in a constant and sustained pattern. Also, pDA is its high hydrophilicity, which brings great beneficial for interface compatibility and cell adhesion. Lai, Min et al. [38] showed that the immobilization of biomolecules on the surface of materials through polydopamine coating on titanium nanotubes effectively promoted osteogenic differentiation of mesenchymal stem cells. It was shown to be a feasible method to effectively deliver osteoinductive signals to guide bone regeneration [31]. Overall, numerous studies have shown that polydopamine-mediated immobilization of growth factor surfaces is effective in tissue engineering applications.

### Characterization of dual growth factor loading

Most of the materials used to fabricate porous microspheres are biodegradable, biocompatible, and nontoxic. Although these porous microspheres used as scaffolds can provide living space for cells and enhance cellular activity to some extent, the lack of bioactivity and limited bone repair capacity still remain a problem [11, 39]. In contrast, nHA/PLGA microspheres made by the airflow shear method have a large specific surface area and high porosity due to their unique surface morphology and can be effectively surface immobilized with growth factors after surface modification by pDA [31].

Recent studies have shown that the combination of BMP-2 and other cytokines can synergistically enhance the osteogenic differentiation and proliferation capacity of bone marrow MSCs, resulting in a more desirable osteointegration capacity of multigrowth factor-modified bone tissue engineering cell carriers [40]. For example, the combination of insulin-like growth factor-1 (IGF-1) and BMP-2 has a superior ability to induce osteogenic differentiation compared to BMP-2 alone, and can stimulate bone regeneration more effectively [41]. Coadministration of insulin-like growth factor-1 (IGF-1) and transforming growth factor-β1 (TGF-β1) promotes bone regeneration [42], and alginate scaffolds loaded with the dual growth factors TGF-β3 and BMP-2 can significantly enhance cellular osteogenic differentiation compared to scaffolds loaded with a single growth factor [43]. bFGF and BMP-2, acting as typical mitogens and morphogens, respectively, are widely used in bone tissue engineering [27]. In a nude mouse model of cranial defects, human bone marrow mesenchymal stem cells were able to differentiate into an osteogenic lineage and promote bone repair under the costimulation with BMP2 and bFGF [44]. As mentioned above, the combined application of multiple growth factors has been shown to produce synergistic effects that may further induce more complex cellular events and interactions, thereby promoting new bone formation [23]. The combined use of bone morphogenetic protein (BMP-2) and basic fibroblast growth factor (bFGF) for osteogenic differentiation of HUMSCs has become a current research hotspot [19, 45]. Since under nonoptimized conditions, the combined delivery of BMP-2 and bFGF instead reduces the osteogenesis of MSCs [24], it is of great importance to enhance the synergistic osteogenic effects of these two growth factors. Parameters such as the concentration and ratio of each growth factor need to be further optimized and adjusted.

**Fig. 2:**
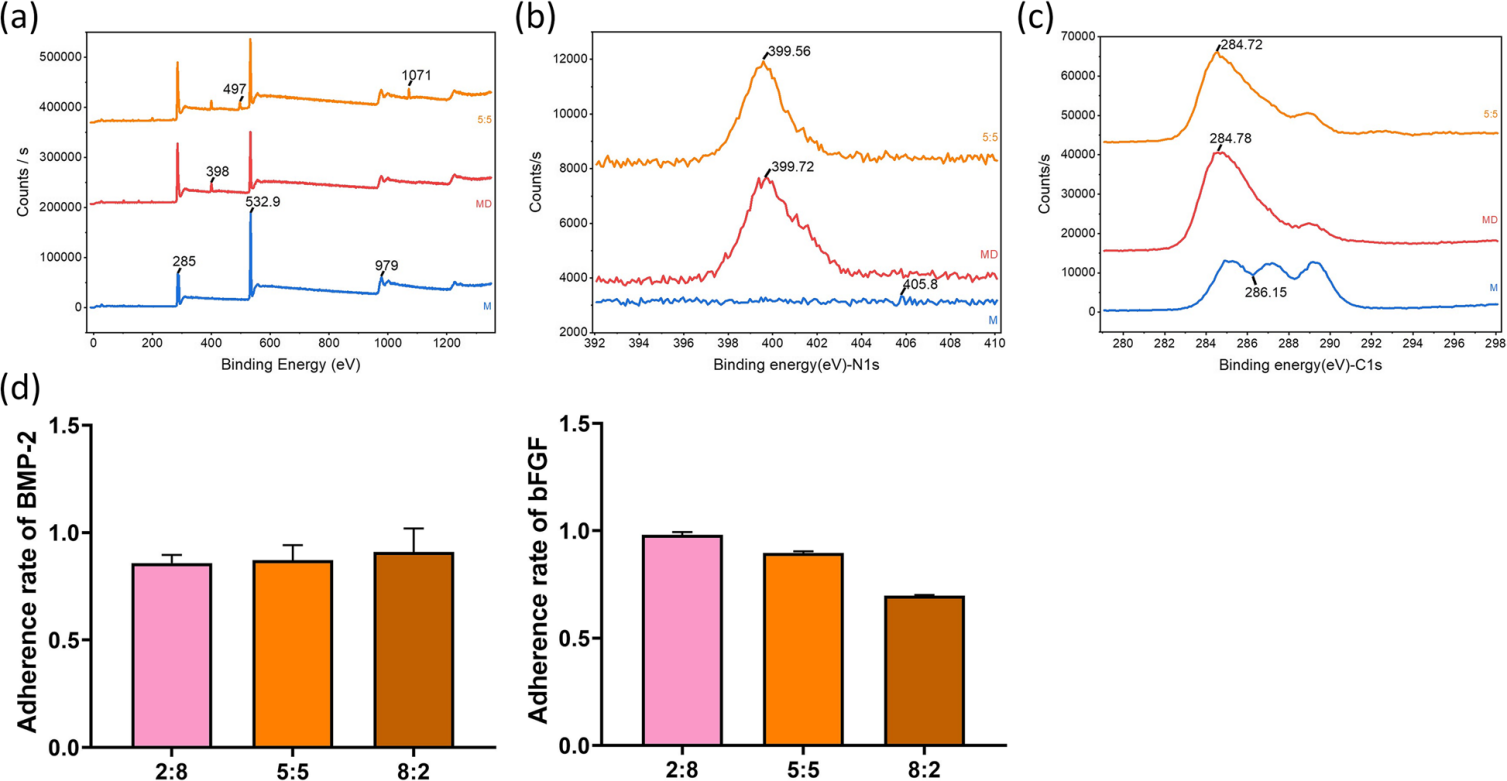
**a** XPS of pDA/nHA/PLGA-GF, pDA/nHA/PLGA, and nHA/PLGA. **b** N1s XPS scan of pDA/nHA/PLGA-GF, pDA/nHA/PLGA, and nHA/PLGA. **c** C1s XPS scan of pDA/nHA/PLGA-GF, pDA/nHA/PLGA, and nHA/PLGA. **d** Adhesion rate of growth factors mediated by pDA coating on the surface of nHA/PLGA composite microcarriers. *p* < 0.05, *n* = 3

In this study, growth factors immobilized on the surface of MD were quantified using ELISA. After treatment with different ratios (2:8, 5:5, 8:2) of BMP-2/bFGF dual growth factor solution, 8.59 ng, 21.80 ng and 36.38 ng of BMP-2 were immobilized on the surface of MD, and the amounts of bFGF immobilized on the surface of MD were 39.26 ng, 22.40 ng and 6.98 ng, respectively. This indicated that the dual growth factor could adhere to the microcarrier surface at approximately the set ratio. The respective adhesion rates of BMP-2/bFGF in the combined BMP-2/bFGF dual growth factor group were also calculated, and the results are shown in Fig. 2a. The high adhesion rates of growth factors on the material surface mediated by the pDA coating indirectly proved that pDA coating for surface modification is an efficient and simple method for introducing adherence to biokines.

To confirm the surface segregation, XPS measurements of the microspheres were performed (Fig. 2b). According to the results, we could assign the signal at 285 eV to C-H and C–C, which was found in all groups. Similarly, the O signal was located at 532.9 eV and 979 eV. In the pDA group, another peak could be found at 398 eV, representing the P = N-P functional group. The existence of N indicated the adherence of pDA. However, 497 eV and 1071 eV in the GF group were attributed to Na. The N1s scan showed peaks at 399.72 eV, 399.56 eV in teh pDA and GF groups, respectively. In C1s scanning, we could assign the signals at 288.7, 286.6 and 284.6 eV to the carboxylic carbon (–CO–O), to the neighboring carbon in chain (C–O) and to the alkyl carbon (C–H) in the nHA/PLGA group. After modification with pDA, the characteristic peaks became extremely sharp at 284.72 eV and 284.78 eV, and the peak binding energy in the medium was covered. This may have been caused by hydrocarbons in pDA, further indicating the modification of the pDA layer.

### In vitro biocompatibility of the modular tissue engineering construct

In this study, we investigated the promoting effect of different ratios of BMP-2 and bFGF on the proliferation and osteogenic differentiation of HUMSCs. By using a combined group of nHA/PLGA-pDA microspheres loaded with complex growth factors, the optimal ratio of BMP-2 and bFGF was evaluated in vitro. The effects of nHA/PLGA-pDA microspheres modified with different ratios of the BMP-2/bFGF dual growth factors on the proliferation of HUMSCs are shown in Fig. 5. The histograms show the ratio of the cell proliferation capacity of HUMSCs after 1, 3 and 7 days of culture on the surface of BMP-2/bFGF dual growth factor-modified samples versus the remaining group of samples. On the first day of incubation, there was no significant difference in the absorbance of the cells in each group. On Day 7, the absorbance values of all groups increased significantly, and the absorbance values of the MF group increased compared with those of the MD group and the MB group, indicating that the surface modification of bFGF significantly enhanced the proliferation ability of HUMSCs while BMP-2 had no significant pro-proliferative ability.

The cell proliferation in the surface-modified dual growth factor group was significantly higher than that in the single growth factor group, indicating that the nHA/PLGA-pDA-loaded BMP-2/bFGF dual growth factor had a synergistic effect in promoting the proliferation of HUMSCs. In addition, the cell proliferation rate was significantly higher in the 5:5 group (both BMP-2/bFGF concentrations were 25 ng/ml) than in the other dual growth factor groups, indicating that the 5:5 BMP-2/bFGF concentration ratio was the optimal ratio for the dual cytokines to synergistically promote cell proliferation.

**Fig. 3:**
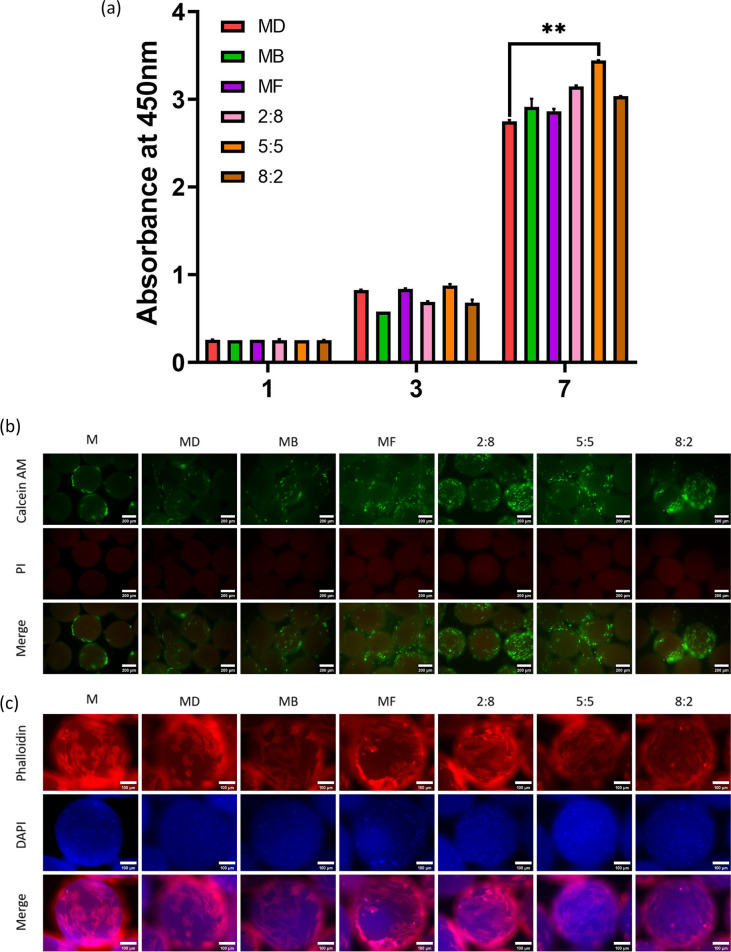
**a** A CCK8 assay was performed to evaluate the proliferation of the HUMSCs cocultured with each group of microspheres for 1, 3, and 7 days. One-way ANOVA with multiple comparison tests was used for statistical analysis (**p < 0.01). **b** Live/dead assay of HUMSCs cultured on each group of microspheres for 4 days. Green staining represented live cells, and red staining represented dead cells. Scale bar: 200 μm. **c** DAPI/phalloidin staining indicated the adhesion of HUMSCs on each group of microspheres for 4 days. Blue staining represents the cell nucleus, and red staining represents the cell cytoskeleton. Scale bar: 100 μm

To further investigate the cell adhesion and distribution in the osteon-like micromodules, calcium xanthophyll AM staining was used, and the corresponding fluorescent images are shown in Fig. 3b. The green areas indicate live cells, and red areas indicate dead cells. In all osteon-like micromodules, almost all cells were stained green, and some cells that did not adhere to the surface of the module formed cell aggregates. With increasing culture time, the outer surface of the nHA/PLGA-pDA microspheres was covered by a fusion layer of HUMSCs. In particular, the outer surface of the micromodules with growth factors immobilized on the surface showed more green sites than the group without cytokine immobilization, indicating that the presence of cytokines led to a more significant promotion of the adhesion, proliferation and spreading of HUMSCs and that the high specific surface area of the nHA/PLGA microcarriers and the adhesion properties of pDA contributed to the attachment and spreading of cells.

The DAPI/phalloidin staining was used to visualize the cell nuclei and cytoskeleton of HUMSCs adhered on microspheres after 4 days (Fig. 3c). The amounts of HUMSCs adhered to the GF-loaded microspheres were slightly higher than those of M and MD, accompanied by a better stretched morphology, larger lamellipodia and closer connection of the cells with each other, especially in the 5:5 group.

### Osteoblastic differentiation on the modular tissue engineering construct

**Fig. 4:**
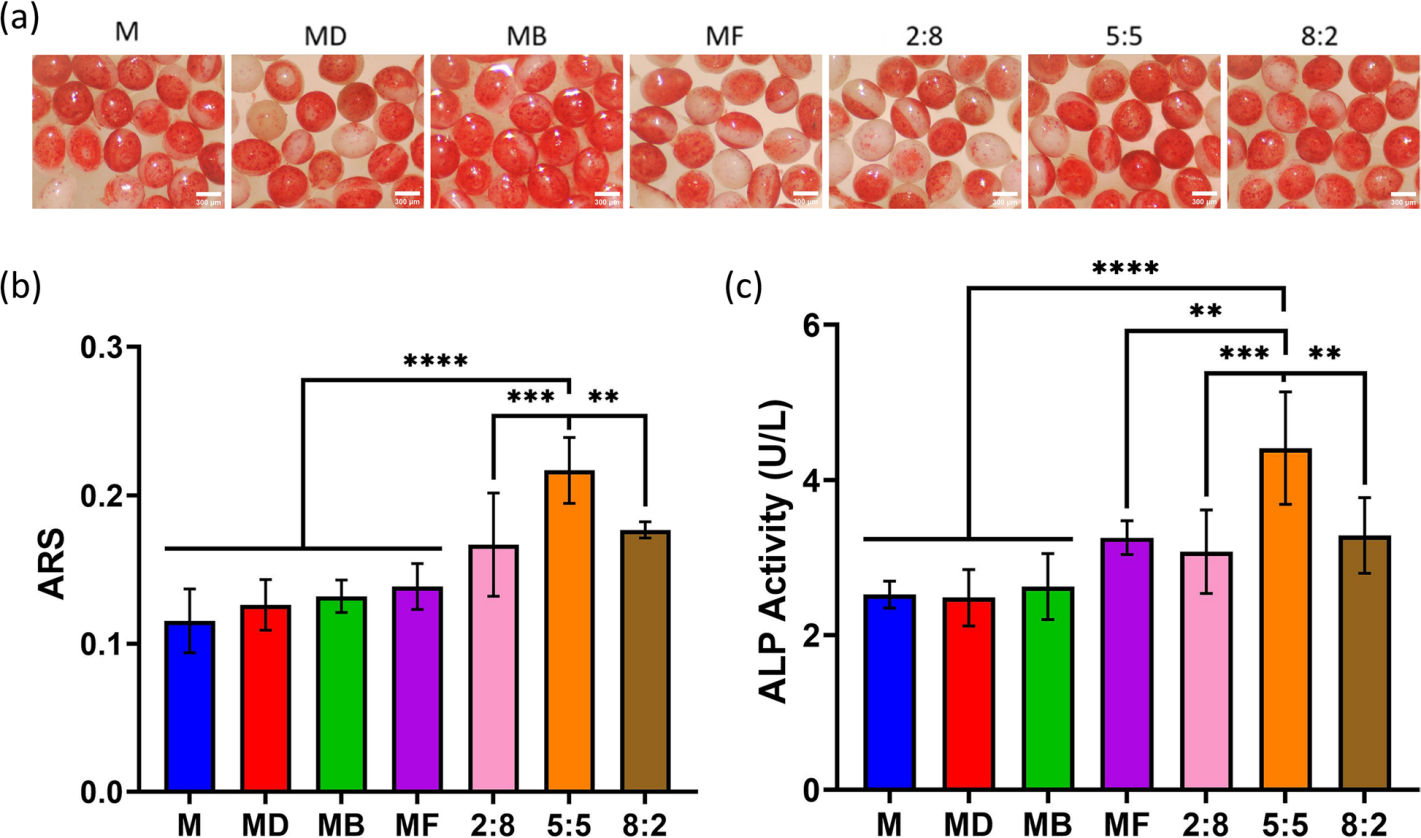
**a** ARS staining micrographs of HUMSCs after 14 days of culture on the surface of nHA/PLGA-pDA microspheres modified with different ratios of BMP-2/bFGF dual growth factors. **b** Quantitative analysis of calcium mineralized nodules in HUMSCs cultured on the surface of nHA/PLGA- microspheres modified with different ratios of BMP-2/bFGF dual growth factor for 14 days. *p* < 0.05, *n* = 3. **c** ALP activity of HUMSCs cultured on the surface of nHA/PLGA- microspheres modified with different ratios of BMP-2/bFGF dual growth factor for 7 days. *p* < 0.05, *n* = 3

Alkaline phosphatase (ALP) plays an extremely important role in the process of osteogenic differentiation of cells, so osteoblast ALP activity is often used as a marker of early osteogenic differentiation. Figure 4c shows the histogram of alkaline phosphatase activity (ALP) of HUMSCs cultured on different sample surfaces for 7 days. ALP activity was enhanced on the surface of the MB group and the MF group relative to the MD group, indicating that surface-modified BMP2 and bFGF could significantly enhance the osteogenic differentiation of HUMSCs. The ALP activity in the combined BMP2/bFGF dual growth factor group was significantly increased compared with that in the single growth factor-modified group, demonstrating that the combination of BMP2 and bFGF induced the differentiation of HUMSCs into osteoblasts better than the single growth factors, and the BMP2/bFGF dual growth factors had a synergistic osteogenic effect. In addition, the sample group with a 5:5 BMP2/bFGF ratio showed significantly enhanced ALP activity relative to the other experimental groups. The results indicate that the optimal ratio of nHA/PLGA-pDA-loaded BMP2 and bFGF dual growth factors synergistically contributing to bone is 5:5.

Alizarin red (ARS) staining was used to examine the ability of HUMSCs to form calcified nodules after 7 days of culture to investigate the ability of different ratios of growth factors to synergistically induce late osteogenic differentiation. Figure 4b shows the quantitative analysis of mineralized nodules of ARS staining after 7 days of culture. After surface modification with dual growth factors, there was a significant increase in calcium nodule mineralization in the compound growth factor groups compared to the other groups. In particular, calcium mineralized nodules were most abundant in the group with a 5:5 ratio of BMP-2/bFGF (Fig. 4a), which was consistent with the ALP results.

### Micro-CT examination of in vivo repair of cranial defects in rats

**Fig. 5:**
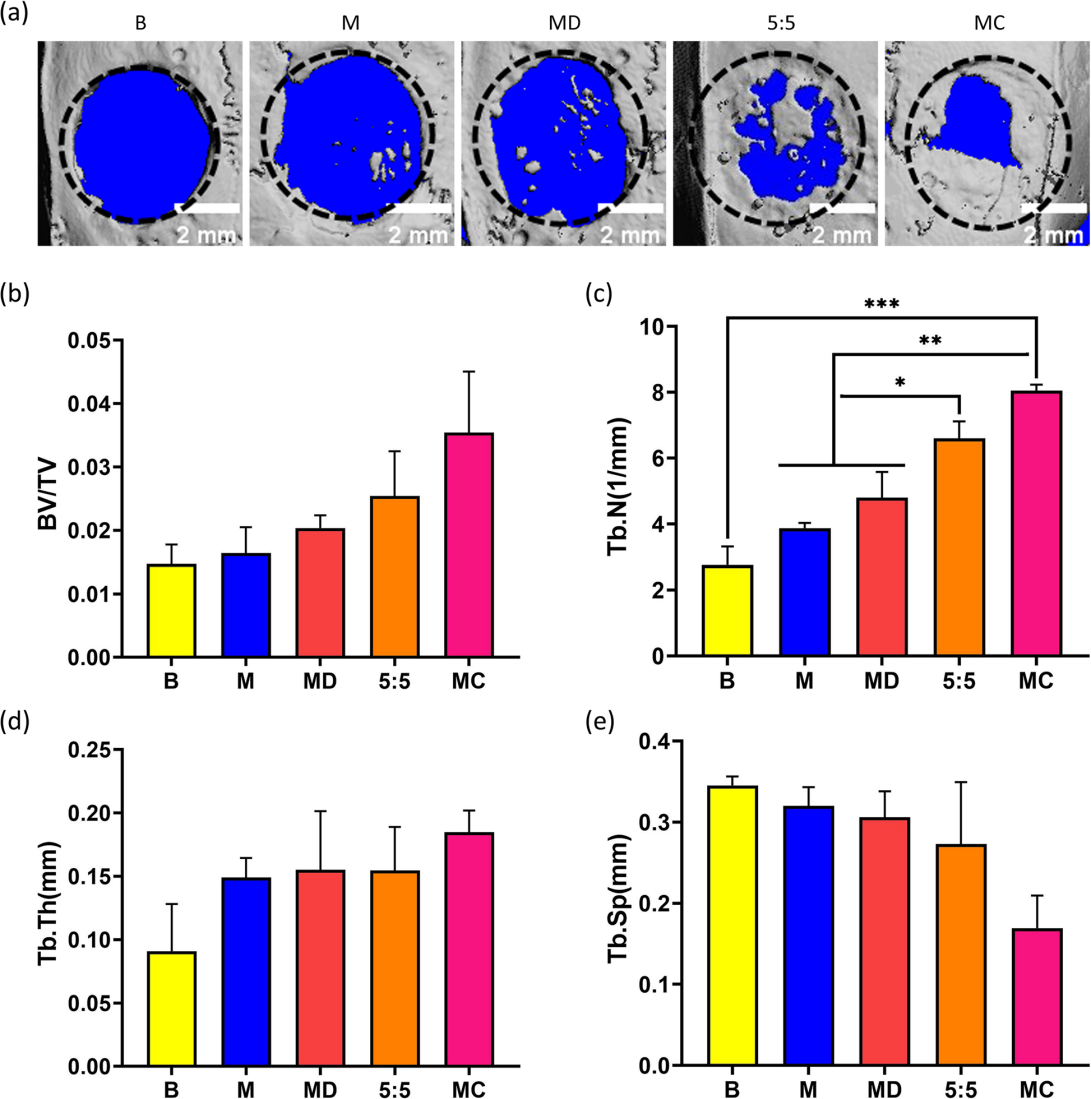
In vivo bone regeneration of composite microspheres after 8 weeks of in vivo implantation. **a** Micro-CT 3D reconstruction of rat skull specimens in each group. **b** BV/TV: bone volume/total volume. **c** Tb.N: Trabecular number. **d** Tb. Th: Trabecular thickness. **e** Tb. Sp: Trabecular separation. One-way ANOVA with a multiple comparison test was used for statistical analysis. *p* < 0.05, *n* = 3

The quality and quantity of newly formed bone can be assessed by evaluating the volumetric properties and geometric parameters of the bone [46]. For this reason, micro-CT is considered to be the gold standard method for evaluating mineral density and three-dimensional microstructure [47]. Figure 5a shows the reconstructed micro-CT images of the defect site implanted with different microcarriers. The size of the 5 mm critical bone defect was found to be as expected in 3D reconstruction images, and the blank group (B) without any treatment was poorly repaired. Most of the defect area remained translucent, and no obvious bone tissue deposition was seen, which can be considered as almost no new bone formation. The bone defect areas filled by microspheres with different surface modification methods showed different degrees of bone repair effects. Among these groups, MC had the best bone repair effect, basically covering the defect site, and the new bone had a certain thickness. In contrast, the M and MD groups without growth factor modification still had more degraded microspheres remaining at the defect site, and the new bone formation was significantly lower than that of the experimental group with growth factor modification. In addition, to further analyze the osteogenic effect of osteogenic micromodules on the bone defect site, the micro-CT results were quantified. The bone volume fraction (BV/TV), bone trabecular number (Tb.N), bone trabecular separation (Tb.Sp) and bone trabecular thickness (Tb.Th) near the defect area were calculated. Among these, BV/TV is the ratio of bone tissue volume to tissue volume, which can directly reflect bone volume. As shown in Fig. 5b, an increase in this value indicates an increase in bone volume, and vice versa. Furthermore, the porous lattice structure formed by bone trabecular junctions has a nonuniform anisotropic arrangement, which can improve the strength of bone. Tb.N, Tb.Sp and Tb.Th are important indicators to evaluate the spatial structure of bone trabeculae. When bone formation was greater than osteolysis, Tb.N and Tb.Th increased and Tb.Sp decreased (Fig. 5c-e). The results showed that the bone-related parameters of the MC group were significantly better than those of the other microcarrier and B groups, exhibiting maximum BV/TV, Tb.N, and Tb.Th and minimum Tb.Sp. These results suggest that the MC group was the most effective in repairing cranial defects and had the greatest ability to promote new bone formation.

### Histological analysis of in vivo repair of cranial defects in rats

To investigate the ability of microtissue-engineered building blocks in vascular network formation and osteogenesis, the implanted microtissue-engineered building blocks were characterized by histological staining.

**Fig. 6:**
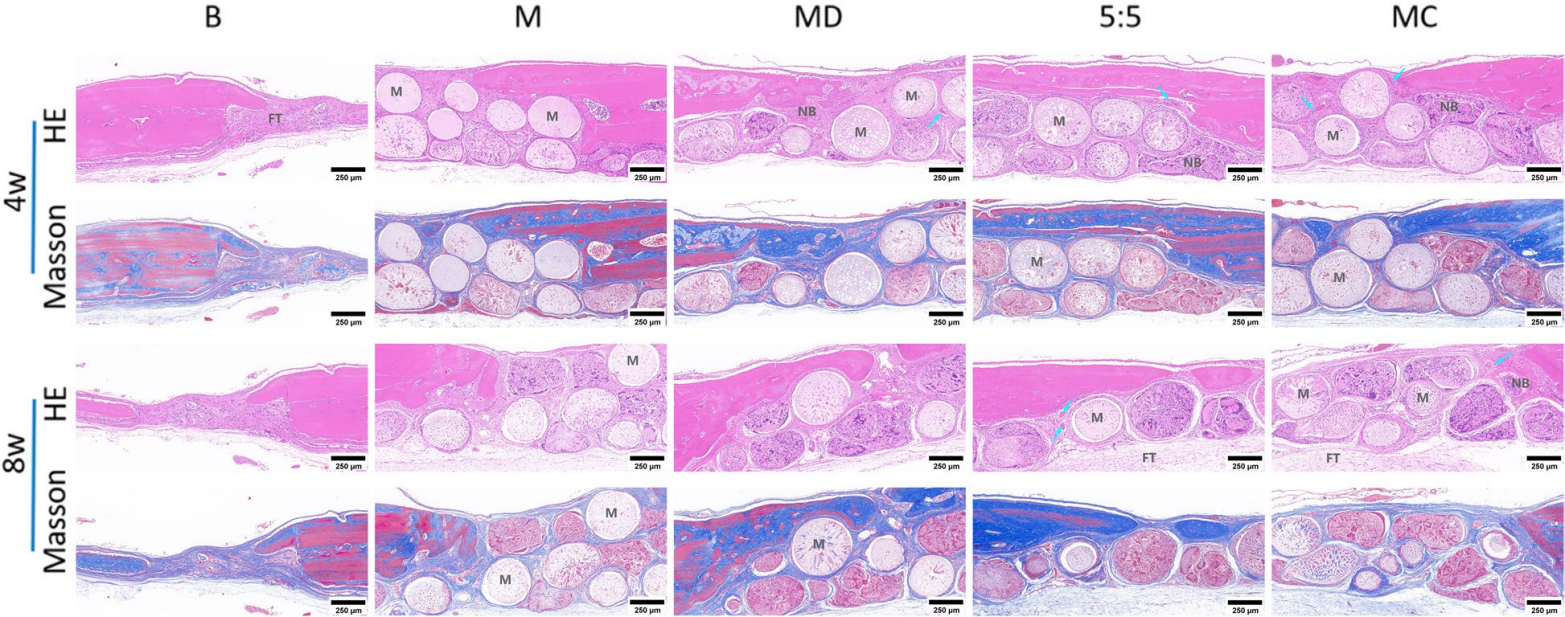
Hematoxylin–eosin (HE) staining and Masson staining of cranial bone defect repair in rats at week 4 and week 8. m: microsphere residue, FT: fibrous tissue, NB: new bone, blue arrow: blood vessel

HE staining (Fig. 6) shows the spatial distribution of cells and the presence of blood vessels in the engineered tissue structures. The cell nuclei appear blue, the bone tissue appears rose-red, and round-like incompletely degraded porous microspheres are distributed in the defect areas. The bone defects in the B group did not show nascent bone bridges but rather dense fibrous tissue. Four weeks after implantation, cells proliferated dramatically and were distributed around the MC and MGF implants. Among all implants, MC had the highest number of cells. Considering that it was due to the presence of cytokine bFGF, which enhanced functional expression in cells, it was able to enhance the proliferation, aggregation and migration behavior of cells around the micromodule. After 8 weeks of implantation, cell proliferation was more pronounced, and all samples showed varying degrees of osteogenic potential due to the addition of nHA to the microspheres. In particular, MC showed the most prominent osteogenic potential, as MC exhibited more pronounced degradation and deformation than M and MD and apparently showed in situ growth of new bone tissue at the damaged microspheres. In addition, a small amount of new bone formation was observed in the MD group with the modified polydopamine coating compared with the M group. No inflammatory cells were observed in any of the groups, indicating that the microscaffolds had good in vivo biocompatibility. Due to the adhesion property of the polydopamine coating, it is not only an ideal material for surface modification of microspheres and further loading of cytokines but also a perfect medium for attachment of materials and bone defects.

In Masson-stained images (Fig. 6), defects in the B group showed little collagen formation, but different degrees of collagen deposition were visible in the defect areas of the implanted microspheres. Consistent with the HE staining results, microspheres undergoing dual growth factor surface modification showed good bone repair ability, with the MC group exhibiting more pronounced collagen tissue deposition. In addition, internal angiogenesis with characteristic erythrocytes was observed (blue arrows). Blood vessels were most abundant within the groups with growth factors. Among them, more new bone could be seen in the MC group, which is consistent with the micro-CT results.

The staining results at week 8 showed more obvious deformation and even fragmentation in the MGF and MC groups than at week 4, indicating that the nHA/PLGA microspheres were biocompatible and had good degradability. Furthermore, a large amount of new bone deposition was observed in situ upon deformation of the microspheres, which is consistent with the micro-CT results. Thus, the osteogenic microunits constructed by using nHA/PLGA microspheres, surface-modified dual growth factors and HUMSC seed cells have excellent osteoinductive ability and can effectively stimulate bone tissue regeneration.

Neoangiogenesis also acts as a necessary step for bone regeneration. In this uexperiment, it was observed that neovascularization was more abundant at the defect in the MGF group. This may be related to the ability of bFGF to effectively stimulate the proliferation of vascular endothelial cells. Therefore, angiogenesis, acts as a beneficial process for bone tissue regeneration, indirectly proving that bFGF is important for promoting bone repair.

Type I collagen is the most abundant bone matrix protein, accounting for 90% of the organic matrix [48]. Its expression and deposition play an important role in bone formation and mineralization [49]. Under ordinary light microscopy, collagen fibers are red, and nuclei are blue. Under polarized light microscopy, collagen fibers have the property of positive uniaxial birefringence of light, which enhances birefringence and improves resolution when combined with Sirius red composite stain, thus distinguishing different types of collagen fibers. Type I collagen fibers appear strong orange‒yellow or bright red, type II collagen fibers appear in various colors, and type III collagen fibers appear green. We mimicked the osteogenic microenvironment by constructing osteon-like micromodules that are suitable for enhancing the biological functions of stem cells as well as osteoblasts to form collagen, which is the first step in the bone mineralization process [50].

**Fig. 7:**
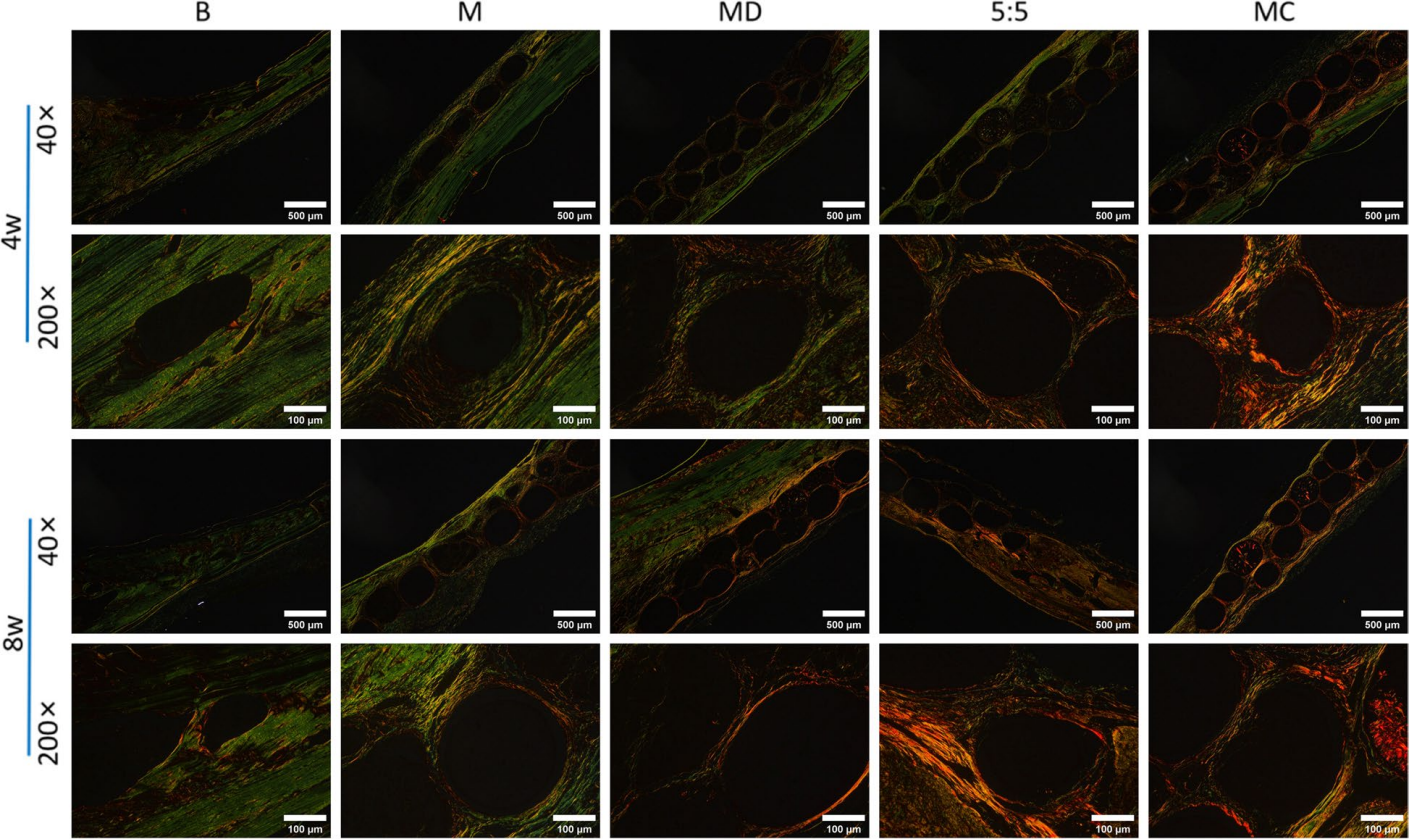
Histological analysis of Sirius red staining after 8 weeks of in vivo implantation. Orange represents type I collagen fibers, and green represents type III collagen fibers

**Fig. 8:**
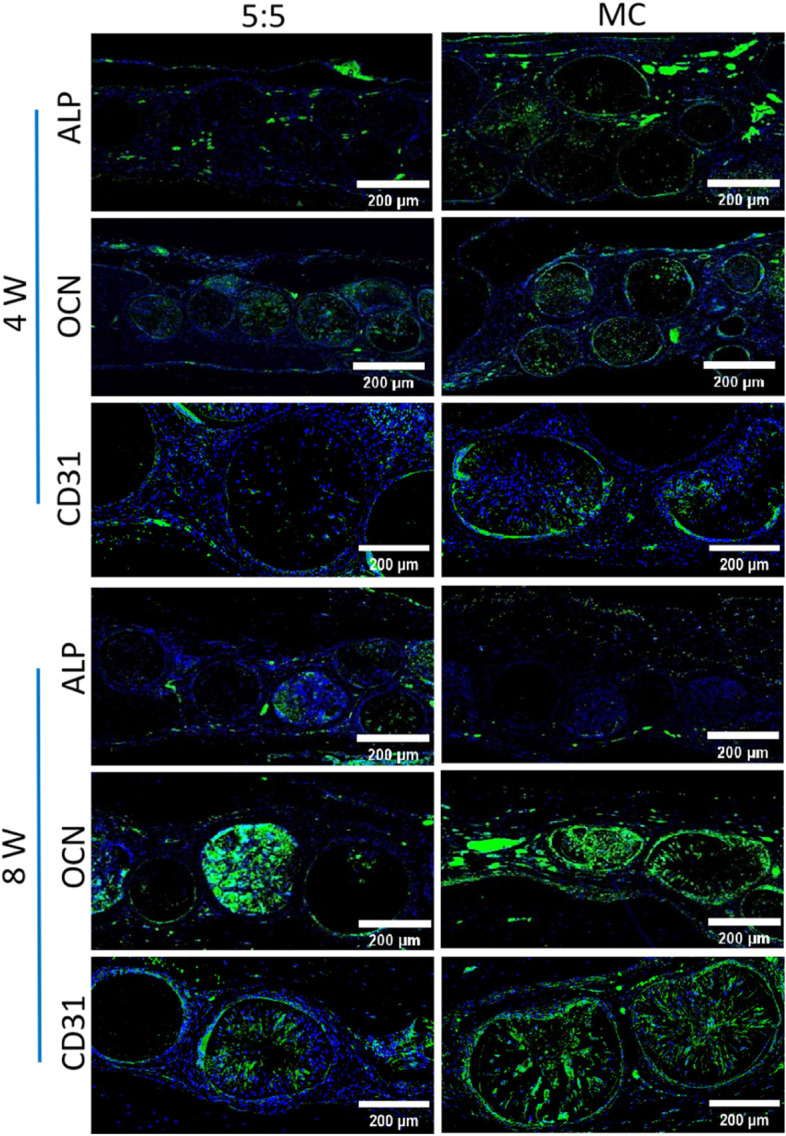
Immunofluorescent staining of ALP, OCN and CD31 was used to assess bone formation and angiogenes. Blue represents the cell nucleus

In the present study, collagen formation analysis was performed on micromodular scaffold structures transplanted in vivo, which showed collagen matrix staining in all tissue structures (Fig. 7). Compared to microsphere controls without growth factors and cell loading, an increasingly widely distributed collagen matrix was present in macroscopic tissue structures assembled from osteogenic micromodules containing HUMSCs and BMP2/bFGF, especially at the edges of the micromodules. Birefringence revealed the presence of highly organized collagen fibers in the constructed micromodule structures, and these results were consistent with the results of HE staining and Masson staining, where type I collagen staining was most evident in the MC structures. The large amount of collagen matrix indicated the formation of a new bone matrix. After 8 weeks of implantation, due to the rapid degradability of the microcarrier and the porous structure inside, the structure provided a site for cell proliferation and migration, leading to the growth of bone tissue into the microspheres. This was also confirmed by Sirius red staining, and ALP and OCN immunofluorescence staining (Fig. 8.), indicating that our constructed osteon-like micromodules had a good ability to promote bone regeneration.

To further assess the induction of osteon-like micromodules for vascular network formation, the neovascular system can be visualized by immunofluorescent staining labeling of the vascular marker CD31 (Fig. 8), also known as platelet/endothelial cell adhesion molecule-1. It is responsible for maintaining and restoring the vascular permeability barrier after disruption of endothelial cell junctions [51]. It can be used to mark neovascular presence [52]. Figure 8 shows that the expression level of CD31 was high, and positive expression of CD31 was seen both inside and at the edges of the tissue structures assembled into micromodules, with a small amount of neovascularization observed at week 4 and more pronounced vascularization at week 8. In the meantime, a more uniform distribution of vessels around the healing area was shown. This suggests that dual treatment with BMP2 and bFGF carried by the micromodule has a significant effect on neovascularization.

Osteogenesis is symbolic of a series of biological events, such as alkaline phosphatase (ALP) expression (an early differentiation marker), osteocalcin (OCN) expression (a late differentiation marker) and ossification [52–54]. OCN is a bone-specific extracellular matrix protein whose expression and synthesis reflect the mature osteoblast phenotype, and ALP plays an important role in osteogenic differentiation. It is often used as a marker of early osteogenic differentiation. In the current study, the effect of osteoid micromodules on bone formation was assessed by measuring the expression of ALP and OCN. The addition of nHA to the microspheres and the osteoinductive properties of the double growth factor resulted in high levels of both proteins in all samples. However, the expression of both molecules was highest in the MC group and became more pronounced in the OCN at week 8 as the time of implantation increased. This evidence suggests that the macroscopic tissue structure formed by the assembly of osteon-like micromodules significantly enhances osteogenic differentiation and bone-associated extracellular matrix deposition in HUMSCs. The formation of more blood vessels contributes to stem cell survival and bone formation in osteon-like tissue.

The results of in vivo experiments showed that MCs constitute the microenvironment for bone defect repair. On the one hand, nHA/PLGA microspheres can be used as osteogenic scaffolding materials to facilitate the filling of irregular bone defect morphology. They support the growth and adhesion of seed cells and help to carry HUMSCs to build osteogenic microunits. On the other hand, the dual growth factors loaded on HUMSCs can induce local blood vessel generation and have a synergistic effect on inducing osteogenic differentiation of stem cells and promoting osteogenesis, thus achieving a good bone defect repair effect.

In this study, HUMSCs showed excellent seed cell properties in bone repair and were proven to have the potential to differentiate into bone tissue. As shown in some cases, HUMSCs piggyback on osteoinductive scaffold material and have the ability of osteogenic differentiation and generation of human-derived bone in vivo [55]. HUMSCs are considered to be the most promising pluripotent stem cells for clinical applications.

### Expression levels of osteogenic-specific genes of HUMSCs in the construct

**Fig. 9:**
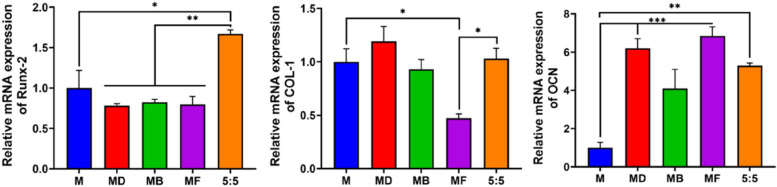
The expression of osteogenesis-related genes (Runx-2, COL-1, OCN) in HUMSCs was evaluated by RT‒qPCR (*n* = 3) 7 days after culture on the surface of the microspheres

The osteogenic differentiation of HUMSCs was directly assessed by measuring the expression levels of related genes, including Runx-2, COL-1 and OCN, by RT‒qPCR analysis (Fig. 9). Runt-related transcription Factor 2 (Runx-2) acts as a specific transcription factor of osteocytes and is regarded as an osteogenic marker gene of early differentiation. Here, the Runx-2 expression level was significantly upregulated in the MGF group, which proved the strong impact of dual growth factors at the early stage of osteogenesis. Osteocalcin (OCN) appears at the late stage of osteoblast differentiation and regulates calcium homeostasis and bone mineralization binding to calcium. COL-1, the most abundant protein in bones, is also osteogenic-related in the bone defect area. Figure 9 shows that there was no apparent advantage of the combination of growth factors according to the relative expression levels of OCN and COL-1. In brief, it was proven that the combination of BMP-2 and bFGF was beneficial, playing a potential role in the early phase of bone formation. Combined with previous results, the application of microspheres with growth factors shows the effect of osteogenesis, and the dual growth factors synergistically promoted osteogenic differentiation. In follow-up studies, long-term RT‒qPCR should be applied to further analyze the expression level of related genes.

### Vascularization analysis of the modular tissue engineering construct

One of the major challenges in tissue engineering is forming blood vessel networks, as osteogenesis and angiogenesis are coordinated processes during lifelong bone formation. Vessel networks supply oxygen and nutrients to the cells. The aim of the current study was to construct osteon-like micromodules by self-carrying stem cells with surface immobilized with dual growth factor (BMP2/bFGF), thus promoting osteogenensis. On the basis of the above experiments, the effect of micromodules on angiogenesis was further investigated.

**Fig. 10:**
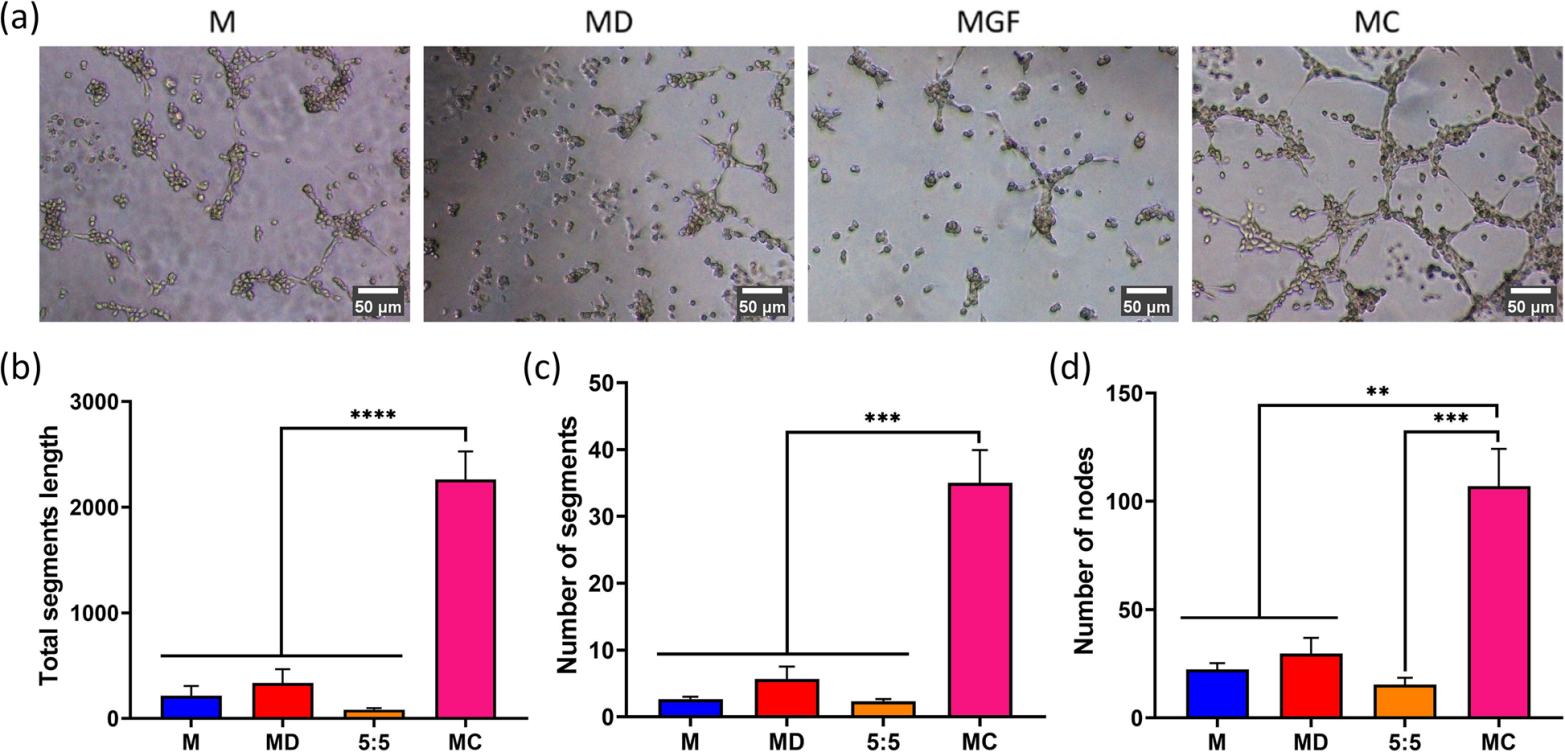
**a** In vitro tube formation by HUVECs conditioned with microspheres. Scale bar: 100 μm. **b**-**d** Quantification of total segment length, number of nodes and number of segments via the angiogenesis tool. One-way ANOVA with a multiple comparison test was used for statistical analysis (**p < 0.01)

Herein, changes in vessel network morphology in response to microspheres were assessed 4 days after stimulation. A custom computational image analysis code in ImageJ was used to characterize and quantify vessel structures. A sharp increase was observed in the MC group in total segment length, number of segments and number of nodes relative to other treated groups, indicating a strong angiogenesis effect of MC micromodules. This was probably due to paracrine mechanisms of HUMSCs through the release of exosomes. For example, HUVEC angiogenic activity was proven to be promoted by upregulation of miR-126-3p in HUMSCs [56]. Such a pattern of slow exosome release somehow ensured the blood supply and accelerated bone reconstruction [57] (Fig. 10).

Bone tissue regeneration is a complex physiological process involving a series of cellular events [58]. The therapeutic effect of bone repair in HUMSCs may be due to cytokines or effective mediators secreted by stem cells in response to surrounding local stimuli. The effective mediators secreted by the cells may be more relevant to the therapeutic properties of MSCs compared to the transdifferentiation of MSCs to host tissue [59]. Ge Yahao and Wang Xinjia [60] revealed a potential molecular mechanism for the osteogenic effects of HUMSC-derived exosomes. In the microenvironment of osteoblast differentiation culture, exosomal miRNAs secreted from osteogenesis-induced HUMSCs are involved in bone development and osteogenic differentiation, such as the MAPK signaling pathway. Among them, hsa-mir-2110 and hsa-mir-328-3p may be the most important osteogenic regulatory miRNAs in exosomes.

It has been shown, observed by tracking the homing of stem cells by luciferase labeling, that the therapeutic effect of stem cell bone repair may be due to the recruitment of endogenous bone progenitor cells. They were stimulated by BMP2, VEGF and other growth factors/cytokines expressed by transplanted MSCs during the initial phase of bone healing [59]. In vivo transplanted MSCs are considered a potential source of growth factors and cytokines required for bone regeneration. In contrast, the osteon-like micromodules constructed in this study improved the low recruitment of osteogenic stem cells and effectively increased osteogenic efficiency by carrying stem cells and dual growth factors, thus achieving full cell coverage of the defect area. bFGF implantation promotes the formation of appropriate vascular and cellular networks around the defect area, effectively improving the nutrient supply and survival rate of stem cells. This, together with the introduction of BMP-2, contributed to the osteogenic differentiation of stem cells in situ as well as the recruitment of bone progenitor cells, thus promoting bone tissue regeneration. These results suggest that the osteon-like micromodules constructed in this study have excellent bone repair ability and provide a new stem cell treatment strategy for bone defect repair.

## Conclusions

In the current study, we fabricated novel osteon-like micromodules for modular tissue engineering. The nHA/PLGA microspheres were prepared as biomicroscaffolds, and human-derived umbilical cord mesenchymal stem cells (HUMSCs) were inoculated onto microcarriers by surface modification of pDA coating and the composite growth factor BMP2/bFGF. They can be used as osteon-like micromodules to develop new engineered osteon-like microunits. The ratio of compound growth factor was also optimized as 5:5 through in vitro experiments. Subsequent in vivo experiments on the repair of cranial defects in rats showed that the mineralization of osteon-like micromodules and new bone formation processes were faster and the bone repair was better with the loaded HUMSCs and compound growth factor. The positive effect was further proven by RT‒qPCR and tube formation assays.

This study showed that the optimized ratio of composite growth factor significantly enhanced the proliferation and differentiation ability of HUMSCs, and the combination of HUMSCs with nHA/PLGA microspheres could be used to successfully construct osteon-like structural microunits. This strategy can effectively promote bone tissue regeneration and has potential applications in stem cell therapy and modular tissue engineering. The construction of bone microunits by replacing BMSCs with HUMSCs is expected to be a new strategy for stem cell therapy for bone defect repair.

## Data Availability

The data that support the fndings of this study are available from the corresponding author, upon reasonable request.
